# Integrating ecophysiology and omics to unlock crop response to drought and herbivory stress

**DOI:** 10.3389/fpls.2024.1500773

**Published:** 2024-11-04

**Authors:** Insha Shafi, Manish Gautam, Rupesh Kariyat

**Affiliations:** Department of Entomology and Plant Pathology, University of Arkansas, Fayetteville, AR, United States

**Keywords:** omics integration, water-stress, herbivore-pressure, resilience, stress priming

## Introduction

Plants are constantly exposed to a diverse spectrum of environmental stressors that typically occur concurrently as multifactorial challenges, both spatially and temporally (Joshi et al., 2024). These stressors include abiotic stresses, such as drought, flooding, salinity, nutrient deficiencies, metal toxicity and extreme temperatures, as well as biotic pressures such as herbivory, pathogen attacks, and inter and intraspecific competition (Porter et al., 2020; González Guzmán et al., 2022). Drought and herbivory are arguably among the most critical factors that limit crop productivity in agricultural settings (Gautam et al., 2024), making it imperative to understand plant responses to their combined effects. While conventional research has tended to examine plant responses to these stressors individually, in both natural and agricultural settings, plants frequently encounter multiple stresses either simultaneously or sequentially, leading to complex interactions with consequences that cascade to multiple trophic levels.

Drought, a major abiotic stress, induces a range of morphophysiological changes in plants, such as reduced leaf area, stomatal closure, and enhanced root growth characterized by increased root biomass, surface area, and root volume (Farooq et al., 2009a). Beyond these immediate responses, drought stress can also prime plants for future stress events, influencing their susceptibility or resistance to subsequent biotic and abiotic challenges (Bilichak et al., 2015; Li et al., 2019; Sallam et al., 2019). Drought stress induced priming can also lead to epigenetic changes in gene expression, creating transcriptional memory that has been found to enhance both plant survival and response to subsequent drought events (Avramova, 2019; Nguyen et al., 2022; Kambona et al., 2023). For instance, in *Arabidopsis*, abiotic stress induces histone demethylation at the promoter of the proline biosynthetic gene Δ1-pyrroline-5-carboxylate synthetase (P5CS), leading to increased P5CS expression and proline accumulation, which assists in tolerating stress. This epigenetic modification remains even after the stress removal, indicating the formation of stress memory that improves tolerance to future stress (Banerjee and Roychoudhury, 2015). Similarly, Sani et al. (2013) (Sani et al., 2013) discovered that sodium ion pre-treated *Arabidopsis* plants gained enhanced drought tolerance through histone modification changes, specifically reduced H3K27me3 levels, which activated the HKT1 gene responsible for salt stress responses (Banerjee and Roychoudhury, 2015). This highlights the critical role of H3K27me3 in somatic stress memory (Kim et al., 2015). Although, drought stress memory, characterized by epigenetic changes in gene expression, has garnered recent attention, the mechanisms through which drought primes plants for future stressors are not well understood (Godwin and Farrona, 2020; Tian et al., 2024).

Similarly, herbivory imposes significant biotic stress on plants, often leading to changes in direct and indirect plant defenses, ecophysiology, defense-fitness trade-offs, production of defensive metabolites, and activation of defense genes, and consequently- affecting trophic interactions (Howe and Jander, 2008; Kariyat et al., 2013; Kersten et al., 2013; Müller et al., 2019; Huang and Huang, 2023). Herbivore-induced plant volatiles (HIPVs), released in response to insect damage or herbivore-cues such as saliva, frass or eggs, serve as signals for predators and parasitoids to locate herbivores at different spatial locations (Kariyat et al., 2012; Ali et al., 2023). Notably, herbivory stress responses can also prime plants to subsequent attacks. For example, feeding by caterpillars on *Arabidopsis* and tomato increased resistance in the subsequent progeny, indicating the presence of transgenerational memory (Van Hulten et al., 2006; Rasmann et al., 2012; Holeski et al., 2012). While existing research on herbivory primarily focuses on plant defenses and their impact on herbivore growth and development (Kariyat et al., 2017, 2018; Tayal et al., 2020; Kaur and Kariyat, 2023), a comprehensive examination in this area should also include physiological responses, such as immune response in herbivores, identification of molecular markers for faster diagnosis, and potential epigenetic modifications that may influence growth and yield traits in subsequent generations (Schmitz et al., 2019).

Despite significant advancements in molecular biology, there remains a lack of a comprehensive molecular understanding of plant responses to abiotic and biotic stresses, when they arrive in tandem. It should be noted that significant progress has been made with techniques like genome sequencing, quantitative trait locus (QTL) mapping which has enabled the identification of key genes involved in drought and herbivory tolerance (Pfalz et al., 2007; Yesudas et al., 2010; Gahlaut et al., 2017; Hassan et al., 2023). And genome wide association studies (GWAS) to identify stress-related traits (Davila Olivas et al., 2017; Thoen et al., 2017; Huang et al., 2024), clustered regularly interspaced short palindromic repeats (CRISPR) for precise genome editing to enhance stress tolerance (Liu et al., 2020; Ghosh and Dey, 2022; Sun et al., 2024), and RNA interference (RNAi) for targeted gene silencing (Li et al., 2024; Zhang et al., 2024), there remains a crucial gap in integrating these molecular tools with ecological interactions. While various biotic and abiotic stressors exist, this manuscript focuses on drought and herbivory as a model due to their synergistic effects, which often intensify plant stress responses (Gautam et al., 2024). By focusing on drought and herbivory, we aim to elucidate how plants manage multiple stresses and propose integrated approaches for studying these complex interactions. This integrated perspective will help bridge the gap between molecular biology and ecological interactions, offering new insights into plant resilience and stress management in natural and agricultural ecosystems.

## Eco-physiological and biochemical approaches for studying drought and herbivory in agricultural crops

Drought and herbivory bring significant changes to the crop eco-physiological responses including root architecture, and physiological parameters (Makbul et al., 2011; Bansal et al., 2013; Kunert et al., 2016; Xu et al., 2018). Reduced stomatal conductance, impaired photosynthesis, and decreased chlorophyll content have been consistently observed under drought stress (de Freitas Bueno et al., 2009; Farooq et al., 2009b). Contrary to the consistent effects under drought, herbivory has differential impacts on plant performance; some studies show negative impact on crop performance while others report positive compensatory effects on photosynthesis and crop growth (Kucharik et al., 2016; Dong et al., 2019; Zheng et al., 2021) with an overall negative impact on crop yield (de Freitas Bueno et al., 2009; Grinnan et al., 2013; Owen et al., 2013; Musser et al., 2022). The eco-physiology of the crops under drought and herbivory are mostly resulted by the biochemical changes in the host crops (Abid et al., 2018; Chávez-Arias et al., 2021; Pati et al., 2023). For example, Pati et al. (2023) reported that the action of several defense related enzymes such as peroxidase, polyphenol oxidase, and catalase was higher in resistant genotypes of rice against brown plant hopper (*Nilaparvata lugens*, Stal). At the same time, Chávez-Arias et al. (2021) also summarized those changes in osmolytes such as proline, glycine, soluble sugars, and ions (K^+^, Na^+^) aid in alleviating the impacts of drought stress in crops like maize and rice.

Biochemical approaches are usually indicated by decline in membrane stability, increased reactive oxygen species (ROS) production, lipid peroxidation and injury to the membranes (Abid et al., 2018) under drought stress. Higher accumulation of proline, free amino acids, and soluble sugars have also been identified as mechanisms of plants for osmotic adjustments under drought stress (Oh and Komatsu, 2015; Mwenye et al., 2016; Du et al., 2020; Chávez-Arias et al., 2021). Similarly, based on the types of crops and herbivores, crops may respond differentially with varying quantities of phenols, soluble proteins, and activities of defense enzymes including peroxidases, and catalases (Pati et al., 2023). Interestingly, production of secondary metabolites in crops such as flavonoids, terpenoids, alkaloids, and glucosinolates are triggered in response to both drought and herbivory (Lin et al., 2023). Besides secondary metabolites, drought and herbivory also alter the volatiles (VOCs) emitted by the crops. For instance, herbivory by *Spodoptera exigua* in potato (*Solanum tuberosum*) under lower water availability induced lower VOCs than under well-watered plants (Vázquez-González et al., 2022). Most importantly, volatile compounds and secondary metabolites induced under drought and herbivory leads to signaling cascades in crops triggering the production of systemic phytohormones (Jogawat et al., 2021).

Phytohormones such as jasmonic acid (JA), salicylic acid (SA), and abscisic acid (ABA) have been studied to explore the possible signaling mechanism for crop responses under drought and herbivory. A decreasing trend was observed for JA and methyl jasmonate (Me-JA) under drought and herbivory stress in soybeans compared to herbivory only treatment, but SA showed a reduction only under drought but not due to insect infestation (Faustino et al., 2021). However, compared to control treatments (no drought or herbivory), JA and Me-JA levels were still upregulated indicating a defensive response under drought and herbivory interaction. Interestingly, under severe drought and simulated herbivory (exogenous application of MeJA), several volatile metabolites including methyl salicylate is strongly induced potentially due to priming under drought stress (Scott et al., 2019). At the same time, under drought stress, ABA has been found to synergize with JA to provide resistance against herbivores (Nguyen et al., 2016). Jogawat et al. (2021) mentioned that changes in level of ethylene (ET) affected growth responses in wheat and drought induced the genes encoding for ET receptors in rice, *Arabidopsis thaliana* and tobacco. Moreover, the expression levels of lipoxygenases (LOX) genes that are involved in the first step of jasmonic acid (JA) biosynthesis were upregulated under drought but was repressed when followed by herbivory in soybeans (Faustino et al., 2021). In a meta-analysis, transcriptomics of major crops such as wheat, maize, and tomato revealed that ET pathway and ABA pathway-related transcription factors are upregulated under drought (Benny et al., 2019). In addition to eco-physiology, biochemical and phytohormonal assessment, examination of several omics and epigenetics approaches can potentially extrapolate the mechanistic understanding of drought and herbivory interaction in crops. The current approaches focused on phenotypic effects, therefore, are mostly limited to few model species, pairwise interactions and lack an integrated approach. Omics approaches allow us to expand our understanding on the underlying mechanisms of drought and herbivory interactions that we still lack and ask more landscape level questions, which is what we propose, an integration of different approaches.

## Redefining drought and herbivory interaction from omics approach

**Figure 1:**
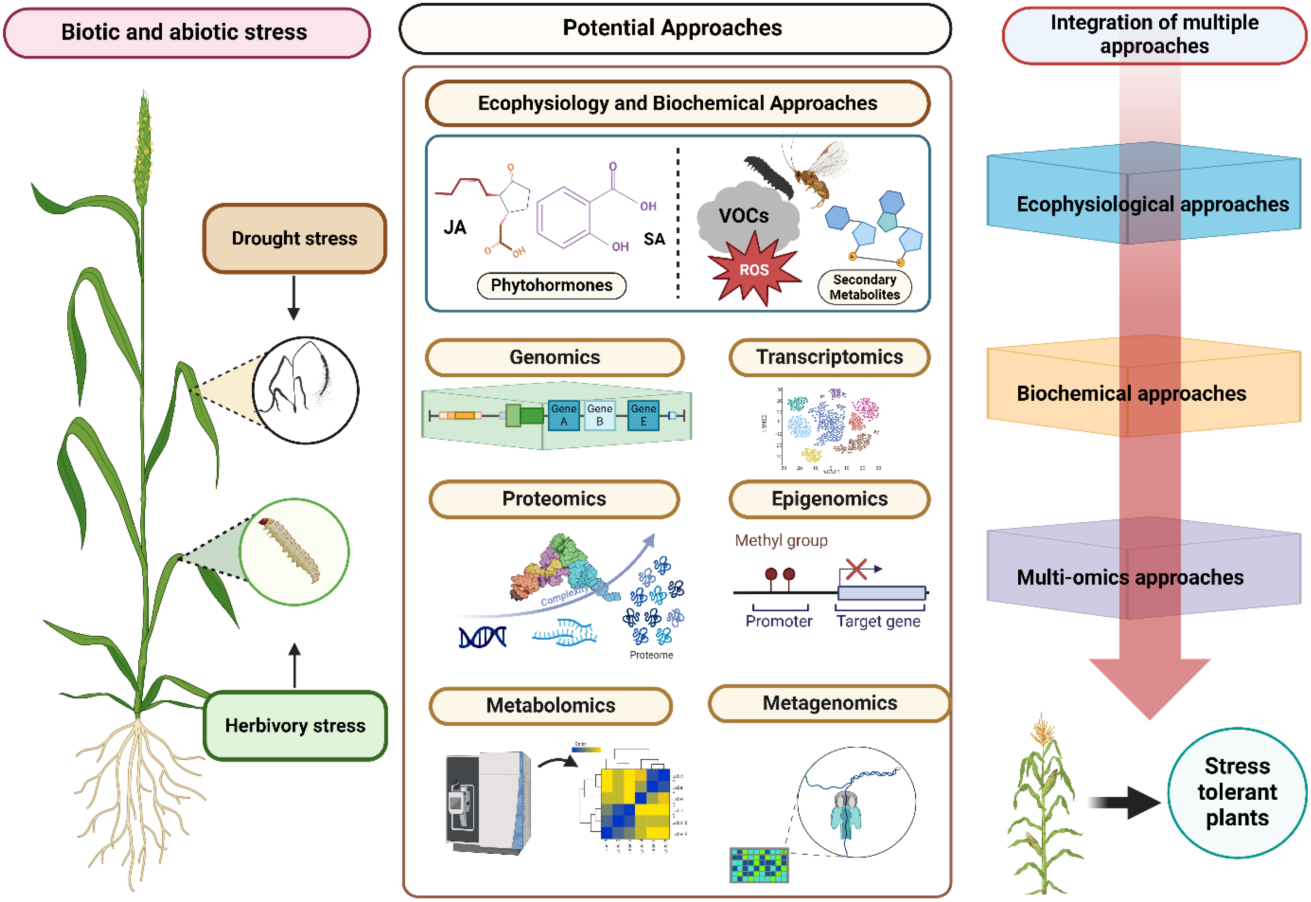
Schematic representation of integration of eco-physiological, biochemical and omics approaches to understand plant environment interactions under biotic and abiotic stress. Eco-physiological approaches involve assessing plant growth, water use efficiency, and stress tolerance, providing valuable insights into how plants adapt to changing environments. Biochemical approaches help in identifying and quantifying stress-related metabolites, enzymes, and hormones, which help elucidate the molecular mechanisms driving stress responses. Omics approaches, including genomics, transcriptomics, proteomics, and metabolomics, generate extensive datasets that uncover the complex regulatory networks and pathways involved in stress adaptation.

Omics approaches help in elucidating the interaction between the environment and genes, characterization and identification of biomarkers and phenotyping (Chávez-Arias et al., 2021). Innovative approaches that combine data from multiple omics layers, such as transcriptomics metabolomics, proteomics, epigenomics, and metagenomics—collectively referred to as panomics - are some of the approaches under ‘Omics’ which have recently become critical for understanding the pathways, genetic and biochemical basis of drought and herbivory interaction in agricultural crops (**Figure 1**).

## Differential expression of genes under drought and herbivory

Transcriptome profiles under simultaneous drought and herbivory stress have been widely used to elucidate their interactive effects on several plants. (Coolen et al., 2016) used RNAseq to explore the transcriptome of Arabidopsis under herbivory when previously exposed to drought stress and found that the transcriptional changes due to caterpillars masked the drought-induced changes that occurred previously. Using exogenous coronatine (COR), (Attaran et al., 2014) found that JA-induced signaling repressed photosynthetic genes but defense genes were induced in *Arabidopsis.* Through a comprehensive RNAseq analysis, it was clear that JA-induced signaling (stress response) redirected the photosynthetic metabolism towards defense responses. Similarly, the transcriptional responses were detected with a significant level of upregulation of GmCDPK genes suggesting their integral roles in soybean-drought-insect interactions (Hettenhausen et al., 2016). In rice, 6,885 transcripts and 238 lncRNAs were detected to be contributing for drought stress response by (Li et al., 2019). Coolen et al. (2016) (Coolen et al., 2016) also reported several unique DEGs expressed under drought compared to herbivory in *Arabidopsis thaliana.* In soybean, the transcriptomic profiles have not been explored much in response to drought and herbivory stress either in sequence or in tandem. It is important to note that majority of such studies have been focused on model species such as *Arabidopsis* and it is imperative to drive future works towards exploring drought and herbivory interaction in non-model agricultural crops that vary in their degree of drought or herbivory tolerance.

## Drought and herbivory interactions drives the changes in metabolome

Analysis of metabolites accumulated in plants under drought stress and herbivory provide a more holistic view on the plant responses towards both stressors. (He et al., 2022) showed that drought stress induced the production of Daidzin which also plays an anti-herbivore role in poplars. An untargeted metabolomics using ultra-performance liquid chromatography-mass spectrometry (UPLC-MS) on the metabolites of wheat under drought revealed increased accumulation of phenolics, thymine and pyrimidine in wheat seedlings which provided further resistance against drought stress (Guo et al., 2020). Likewise, metabolomics study in maize has allowed us to identify indole, terpenoids, and green leaf volatiles [(Z)-3-hexanal and (Z)-3-hexen1-ol] as primary secondary metabolites involved in defense against herbivores (Chávez-Arias et al., 2021). Compared to other ‘Omics’ tools, metabolomics seems to be more effectively utilized by researchers when exploring drought and herbivory. However, comprehensive understanding of drought and herbivory interaction in agricultural crops using metabolomics by itself is difficult due to the involvement of myriads of biotic and abiotic factors other than drought and herbivores themselves.

## Overlapping drought and herbivory stressors can alter protein expression

Proteomic approaches elucidate the role of genes related to a specific protein associated with stress, providing a snapshot of the proteome of crop under biotic or abiotic stress (Bhadauria et al., 2010; Ahmed et al., 2024). Although many studies have focused on the individual impacts of drought or herbivory on the proteomic composition of different crops, very few studies have addressed the combined effects of these stressors on crop proteomes (Zhang et al., 2010; Li et al., 2014; Gu et al., 2020). Verdugo et al. (2015) (Verdugo et al., 2015) found that *Myzus persicae*; (Green peach aphids) on drought-stressed susceptible plants showed higher protein expression levels compared to those on well-watered susceptible plants. A study by Dworak et al. (2016) (Dworak et al., 2016) highlights the maize leaf proteome responses to soil drought and two-spotted spider mite (*Tetranychus urticae*) stresses applied separately and concurrently. The findings revealed that the protein carbonylation level, a key marker of oxidative damage, increased with both soil drought and mite feeding when applied separately. However, when these stressors occurred simultaneously, there was a decrease in the protein carbonylation level, indicative of a unique response when the stresses overlap. This underscores the complexity of the interplay between different stressors and their impact on the proteomic profiles of crops, emphasizing the need for thorough research to understand the underlying mechanisms governing these interactions.

## Drought and herbivory induce epigenetic modifications

Epigenetics plays a pivotal role in deciphering how plants respond to biotic and abiotic stresses, offering unique insights into the molecular mechanisms underlying stress adaptation (Mladenov et al., 2021). Epigenetic changes induced by biotic stressors like bacteria, fungi, or insect herbivores can be passed down to offspring, resulting in transgenerational priming (Holeski et al., 2012; Harris et al., 2023). Recent studies have shown that inherited epigenetic modifications can lead to acquired resistance patterns that persist for multiple generations following exposure to biotic stressors (Stassen et al., 2018). Specifically, alterations in DNA methylation, particularly in the CG context, have been observed in response to stress (Huang and Jin, 2022). Some other studies on Thale cress (*Arabidopsis* sp.) and rice (*Oryza* sp.) reveal that long-term stresses like drought-induced changes in DNA methylation can be transferred together with stress memory and improved stress tolerance to multiple generations (Wang et al., 2011; Zheng et al., 2017). Using epigenetic recombinant inbred lines (epiRILs) by (Furci et al., 2019) from a ddm1 mutant crossed with wild-type plants, a screening for downy mildew pathogen *Hyaloperonospora arabidopsidis* (Hpa) -resistant *Arabidopsis* revealed enhanced defense responses and no growth impairments post-infection, indicating defense priming. Transcriptome and DNA methylome analysis showed that priming defense genes across the genome provides lasting, inheritable disease resistance, indicating a crucial role for epigenetic responses in transgenerational acquired resistance in *Arabidopsis*. Hence, by studying the epigenetic landscape of plants under biotic stress conditions, researchers can identify key epigenetic signatures associated with defense responses and priming mechanisms.

## Meta-genomics reveal microbial diversity under drought and herbivory

Drought and herbivory have a broader impact on multi-tropic level interactions in agricultural settings— microbial communities that could potentially directly/indirectly impact the host crop and herbivore performance are often overlooked in most of the studies. Through metagenomics analysis, (Dai et al., 2019) found that several bacteria such as Actinobacteria, and Acidobacteria significantly increased in peanut rhizosphere during drought stress. On the other hand, metagenomics along with metabolomic profile analysis in oilseed rape (*Brassica napus*) under root herbivory by cabbage root fly (*Delia radicum*) revealed significant increment in the abundances of bacterial genera like *Bacillus, Pseudomonas*, and *Stenotrophomonas* (Ourry et al., 2018). It is evident that meta-genomics in combination with other ‘Omics’ approaches can be effective in obtaining a holistic understanding of drought and herbivory interaction in crops. However, to the date, no metagenomics analysis has been conducted to unravel the interaction of these two stressors in terms of both host plants and herbivores and therefore require extensive work in the future.

## Conclusion and future directions

The integration of multi-omics approaches in plant research presents a transformative opportunity to deeply explore the intricate cellular responses that underpin stress tolerance mechanisms. By integrating data from diverse omics pipelines, such as transcriptomics, proteomics, epigenomics and metabolomics, researchers gain a comprehensive understanding of how plants respond to and manage drought and herbivory. This integration allows for the identification of key regulatory networks, biomarkers, and candidate genes that differentiate between stress-tolerant and sensitive plants, providing valuable insights for breeding resilient crop varieties. Moreover, incorporating physiological studies and phytohormone signaling pathways into this multi-omics framework will further enhance our understanding of the complex interactions between plants and their environment. These approaches will elucidate how changes in physiology and hormonal signaling contribute to plant resilience under combined stress conditions. Thus, the integration of multi-omics data in plant research, augmented by physiological and phytohormonal insights, will not only deepen our understanding of stress responses and adaptive mechanisms but also pave the way for the development of resilient crop varieties that can thrive in changing environmental conditions. By merging advanced technologies with traditional breeding methods, researchers can fully harness the potential of omics-assisted breeding for ensuring food security and sustainability in agriculture.
